# Sex differences in associations of socioemotional dispositions measured in childhood and adolescence with brain white matter microstructure 12 years later

**DOI:** 10.1017/pen.2020.3

**Published:** 2020-05-13

**Authors:** Benjamin B. Lahey, Kendra E. Hinton, Francisco Calvache Meyer, Victoria Villalta-Gil, Carol A. Van Hulle, Brooks Applegate, Xiaochan Yang, David H. Zald

**Affiliations:** 1 Department of Public Health Sciences, University of Chicago, Chicago, IL, USA; 2 Department of Psychological Sciences, Vanderbilt University, Nashville, TN, USA; 3 School of Medicine and Public Health, University of Wisconsin, Madison, WI, USA; 4 Department of Educational Leadership, Research, and Technology, Western Michigan University, Kalamazoo, MI, USA

**Keywords:** Dispositions, Fractional anisotropy, Psychopathology, Sex differences

## Abstract

Predictive associations were estimated between socioemotional dispositions measured at 10–17 years using the Child and Adolescent Dispositions Scale (CADS) and future individual differences in white matter microstructure measured at 22–31 years of age. Participants were 410 twins (48.3% monozygotic) selected for later neuroimaging by oversampling on risk for psychopathology from a representative sample of child and adolescent twins. Controlling for demographic covariates and total intracranial volume (TICV), each CADS disposition (negative emotionality, prosociality, and daring) rated by one of the informants (parent or youth) significantly predicted global fractional anisotropy (FA) averaged across the major white matter tracts in brain in adulthood, but did so through significant interactions with sex after false discovery rate (FDR) correction. In females, each 1 SD difference in greater parent-rated prosociality was associated with 0.43 SD greater FA (*p* < 0.0008). In males, each 1 SD difference in greater parent-rated daring was associated with 0.24 SD lower FA (*p* < 0.0008), and each 1 SD difference in greater youth-rated negative emotionality was associated with 0.18 SD greater average FA (*p* < 0.0040). These findings suggest that CADS dispositions are associated with FA, but associations differ by sex. Exploratory analyses suggest that FA may mediate the associations between dispositions and psychopathology in some cases. These associations over 12 years could reflect enduring brain–behavior associations in spite of transactions with the environment, but could equally reflect processes in which dispositional differences in behavior influence the development of white matter. Future longitudinal studies are needed to resolve the causal nature of these sex-moderated associations.

Individual differences in quantitative dispositional traits of temperament and personality are of considerable public health importance because they are substantially correlated with current and future psychopathology and maladaptive functioning in many areas of adult life (Krueger & Markon, 2011; Lahey, 2009; Widiger, 2011). Developmental theorists have posited that individual differences in such dispositions are reliably measured in childhood and predict the likelihood of developing adaptive functioning and psychopathology (Caspi, Henry, McGee, Moffitt, & Silva, 1995; Craske, Poulton, Tsao, & Plotkin, 2001; Forbes, Rapee, Camberis, & McMahon, 2017; Hirshfeld-Becker et al., 2008; Honomichl & Donnellan, 2012; Krueger, Caspi, Moffitt, Silva, & McGee, 1996; Moffitt et al., 2011; Muris & Ollendick, 2005; Nigg, 2006; Song, Waller, Hyde, & Olson, 2016; Tackett, 2006; Thomas & Chess, 1957; Volbrecht & Goldsmith, 2010). Predictive associations between dispositions and psychopathology and functional outcomes may result from individual differences in child dispositions forming the behavioral basis for the development of psychopathology, and/or transacting with the individual’s environments to both (1) influence the likelihood of adaptive and maladaptive experiences and (2) moderate the child’s responses to such experiences (Bell, 1977; Belsky & Pluess, 2009; Sameroff, 2009). This transactional hypothesis has received support from studies that have, for example, found that child temperament interacts with parenting styles (Overbeek, 2017; Slagt, Dubas, Dekovic, & van Aken, 2016), stress (Schermerhorn et al., 2013), and family transitions (Ruschena, Prior, Sanson, & Smart, 2005) in predicting future psychopathology.

In this study, three dispositions were measured at 10–17 years of age using parent- and youth-completed forms of the Child and Adolescent Dispositions Scale (CADS) (Lahey, Applegate et al., 2008; Lahey, Rathouz, Applegate, Tackett, & Waldman, 2010). The CADS differs from general-purpose temperament and personality scales because it was developed specifically to study associations between dispositions and psychopathology. Therefore, to avoid item contamination in estimating correlations with psychopathology, synonyms and antonyms of symptoms of psychopathology were excluded from the CADS item pool (Lahey, Applegate et al., 2008; Lahey et al., 2010). As a result, the CADS differs from other quantitatively derived dimensional measures of dispositions in two ways. First, although some dispositions, such as negative emotionality, are defined in the CADS that are also measured in general-purpose scales, the items that define the CADS scales do not include synonyms and antonyms of symptoms of psychopathology. Second, the CADS intentionally does not measure dispositions that are particularly challenging to disentangle from psychopathological characteristics. For example, no attempt was made to define a dimension of effortful control (Simonds, Kieras, Rueda, & Rothbart, 2007) in the CADS because the items typically used to define that dimension are antonyms of the symptoms of attention-deficit/hyperactivity disorder (ADHD). Similarly, positive emotionality is not measured in the CADS because many defining items are antonyms of dysphoria and anhedonia.

Factor analyses of the CADS item pool defined three largely orthogonal factors that were nearly identical for parent and youth item ratings (Lahey, Applegate et al., 2008; Lahey et al., 2010; Mathesius, Lussier, & Corrado, 2017):

1. CADS *negative emotionality* is defined by items assessing frequent and intense negative emotional responses to frustrations, loss, and threats. In spite of the absence of symptom-like items, the scale is conceptually similar to both the negative affectivity factor of the Children’s Behavior Questionnaire (Rothbart, Ahadi, Hershey, & Fisher, 2001) and the five-factor model trait of neuroticism, which is known to be correlated with well-being and essentially every form of psychopathology across the lifespan (Gale, Booth, Mottus, Kuh, & Deary, 2013; Lahey, 2009; Widiger & Oltmanns, 2017).
2. The CADS *prosociality* scale quantifies caring about the welfare of others, spontaneous helping, attempting to please them, and experiencing guilt over misbehaviors. Prosociality is a widely studied construct under several different names (Frick, Ray, Thornton, & Kahn, 2014b; Goodman, 1997; Hare, 2017; Knafo-Noam, Uzefovsky, Israel, Davidov, & Zahn-Waxler, 2015).
3. Children rated high on the *daring* scale find intense and risky situations to be attractive and rewarding. Daring is closely related to the constructs of sensation seeking (Russo et al., 1993; Steinberg et al., 2008; Zuckerman & Aluja, 2015) and low harm avoidance (Luby, Svrakic, McCallum, Przybeck, & Cloninger, 1999).

In spite of the differences between the CADS and other empirically defined dimensional measures of dispositions that were not designed to study associations with dimensions of psychopathology without item contamination, there is emerging evidence on how the CADS lines up with those scales. In particular, youth-rated CADS negative emotionality was found to be moderately correlated (*r* = 48, *p* < .0001) with neuroticism measured by the NEO-FFI (Costa & McCrae, 1992) and to be significantly but modestly inversely correlated with daring and agreeableness at age 12 years (Lahey et al., 2010). This raises the possibility that CADS negative emotionality will prove to be correlated with the five-factor model stability metatrait (DeYoung, 2006; DeYoung, Peterson, & Higgins, 2002; Wright, Creswell, Flory, Muldoon, & Manuck, 2019). The item content of CADS prosociality is essentially identical to that of measures of dispositional sympathy (Murphy, Shepard, Eisenberg, Fabes, & Guthrie, 1999) and the inverse of the callousness factor of callous-unemotional traits (Frick, Ray, Thornton, & Kahn, 2014a; Waldman et al., 2011). In addition, the item content of CADS prosociality is very similar to a measure of empathic concern (FeldmanHall, Dalgleish, Evans, & Mobbs, 2015; Parkinson & Wheatley, 2014) and a measure of empathizing versus systematizing (Baron-Cohen, 2009; Takeuchi et al., 2013).

Additionally, CADS negative emotionality shares phenotypic and genetic variance with both internalizing and externalizing psychopathology defined in a correlated factors model (Mikolajewski, Allan, Hart, Lonigan, & Taylor, 2013a). Furthermore, CADS daring is inversely associated with anxiety within and across informants (Lahey, Applegate et al., 2008).

A substantial number of studies demonstrate that the CADS dispositional dimensions are robustly correlated with psychopathology at the phenotypic level, in spite of the lack of item contamination. In previous cross-sectional studies, CADS negative emotionality has been found to correlate with both externalizing and internalizing psychopathology (Bai & Lee, 2017; Lahey, Applegate et al., 2008; Lahey et al., 2010; Mikolajewski, Hart, & Taylor, 2019; Taylor, Allan, Mikolajewski, & Hart, 2013). In addition, in a cross-sectional study of the general (p) factor of psychopathology (Caspi et al., 2014; Lahey et al., 2012; Lahey, Krueger, Rathouz, Waldman, & Zald, 2017) in children and adolescents, CADS negative emotionality was robustly correlated with the general factor (Tackett et al., 2013). Prospectively, antisocial and high-risk behaviors during adolescence and adulthood are predicted by greater negative emotionality and daring and inversely predicted by prosociality rated during childhood and adolescence (Lahey, Class et al., 2018; Shaw, Hyde, & Brennan, 2012; Sitnick, Brennan, Forbes, & Shaw, 2014; Sitnick, Shaw, & Hyde, 2014; Trentacosta, Hyde, Shaw, & Cheong, 2009). In longitudinal analyses based on the Tennessee Twins Study (TTS), parent-rated negative emotionality at 10–17 years of age predicted antisocial personality disorder (APD) (Lahey, Class et al., 2018) and predicted the general factor of psychopathology based on self-reported symptoms at 23–31 years, whereas parent-rated prosociality and daring predicted the specific externalizing psychopathology factor (Class et al., 2019).

Estimates of the heritability of the parent-rated CADS dimensions based on the full Wave 1 sample of 2,000 pairs of twins in the TTS were 43% for prosociality, 53% for negative emotionality, and 62% for daring (Waldman et al., 2011). Furthermore, all three CADS dimensions have been found to share substantial genetic variance with concurrent conduct disorder (Waldman et al., 2011) and broadly defined externalizing psychopathology (Taylor et al., 2013), and CADS negative emotionality shares substantial genetic variance with internalizing psychopathology (Mikolajewski, Allan, Hart, Lonigan, & Taylor, 2013b; Tackett, Waldman, Van Hulle, & Lahey, 2011) and the general factor of psychopathology (Tackett et al., 2013).

Given the theoretical and social importance of dispositional traits in childhood and adolescence, studies are needed that advance their understanding at multiple levels of analysis. Consistent with the goals and methods of the burgeoning field of personality neuroscience (DeYoung et al., 2010), we examined prospective associations between individual differences in the three CADS dispositions and an aspect of brain structure with implications for the organized and efficient functioning of the brain. Diffusion tensor imaging (DTI) quantifies variations in indices hypothesized to reflect the microstructural integrity of white matter tracts that connect distributed brain regions (Thomason & Thompson, 2011). Fractional anisotropy (FA) is thought to index the microstructural integrity of myelinated neurons by quantifying the extent to which diffusing water molecules in white matter tracts move in a single direction rather in random directions. Radial diffusivity (RD) reflects the transverse direction of diffusion, which is more constrained by greater myelination. Axial diffusivity (AD) is believed to quantify the rate of longitudinal diffusion along axons (Thomason & Thompson, 2011). The results of previous studies of associations between dispositional traits and indices of white matter integrity have been inconsistent, perhaps due to variations in the size and representativeness of the samples. Nonetheless, a meta-analysis of previous studies of neuroticism indicates that there may be inverse associations between negative emotionality and FA in a broad range of fibers across the brain (Mincic, 2015).

Although the structural and functional neural correlates of prosocial behavior have been reported previously (Zaki & Ochsner, 2012), little attention has been paid to associations with white matter integrity. Nonetheless, an inverse relation between FA in the superior longitudinal fasciculus (SLF) and empathizing has been reported in 567 young adults (Takeuchi et al., 2013), and a small study of young adults found FA to be positively correlated with empathic concern in multiple regions including the SLF, forceps minor, and the inferior fronto-occipital fasiculus (Parkinson & Wheatley, 2014). Furthermore, a number of studies of mostly small and unrepresentative samples assessed associations between white matter integrity and callousness among samples of antisocial youth and adults with mixed results (Waller, Dotterer, Murray, Maxwell, & Hyde, 2017). Little is known about correlations of daring with white matter integrity, but lower harm avoidance has been found to be associated with greater mean skeleton FA in middle aged and older adults (Westlye, Bjornebekk, Grydeland, Fjell, & Walhovd, 2011).

In this paper, we report the results of the first prospective study of predictive associations between dispositions rated in childhood and adolescence and variations in white matter microstructural integrity measured by DTI during early adulthood. This design will provide information on the extent to which such associations are enduring over time. Importantly, we controlled sex of the participants and tested sex-by-disposition interactions in the present analyses for three reasons:

1. There is clear evidence of greater mean and variability in FA across the brain in males than females in the population (Ritchie et al., 2018; Tamnes, Roalf, Goddings, & Lebel, 2018; van Hemmen et al., 2017).
2. There is evidence that dispositions are differentially associated with psychopathology in the sexes (Zinbarg et al., 2010) and evidence of sex differences in the association of measures of white matter integrity psychopathology, including alcohol use disorder (Sawyer et al., 2018), schizophrenia (Hawco et al., 2017; Shahab et al., 2018), and autism spectrum disorder (Zeestraten et al., 2017).
3. Most importantly, there is evidence that socioemotional dispositions are differentially associated in females and males with brain structure and function (Sutin, Beason-Held, Dotson, Resnick, & Costa, 2010; Zeestraten et al., 2017). This includes sex-by-disposition interactions in associations of indices of white matter integrity with empathizing (Chou, Cheng, Chen, Lin, & Chu, 2011) and trait anxiety (Kim et al., 2016; Montag, Reuter, Weber, Markett, & Schoene-Bake, 2012).

## 1. Method

Participants were selected from the Wave 1 of the TTS (Lahey, Rathouz et al., 2008) for the Wave 2 evaluation 10–15 years (median = 12 years) later.

### 1.1 Wave 1 sample and measure of dispositions

The Wave 1 sample is representative of 6–17-year-old twins in Tennessee’s five metropolitan statistical areas (MSAs) in 2000–2001. The Tennessee Department of Health identified all twin pairs born in Tennessee in the eligible age range; 2431 twin pairs were eliminated because they lived outside an MSA. A random sample was selected from the remaining families, stratified by age and geographic subareas, proportional to the number of families. Of 4012 selected households, 3592 (89.5%) were located and screened, with 2646 of screened families being eligible (coresidence with the caretaker at least half time during the past 6 months and twins and caretakers spoke English). Interviews were completed with 2,063 adult caretakers (90.8% biological mothers), with a 70% response rate. When caretakers were interviewed, 98% of both twins were interviewed. After excluding pairs in which either twin had been given a diagnosis of autism, psychosis, or seizure disorder, the sample consisted of 3,990 twins in 1,995 complete pairs. Caretakers classified 71% of the twins as non-Hispanic white, 24% African American, 2% as Hispanic, and 3% as other groups. There were no missing data on demographic variables, except that maternal education for two mothers was imputed from the mean of non-missing values on that variable.

The CADS is a reliable and well-validated measure of three socioemotional dispositions (Lahey, Applegate et al., 2008; Lahey et al., 2010). Items are rated on a 1 (not at all) to 4 (very much) response scale in interviews, separately by parents and youth. The CADS was originally developed to test the hypothesis that antisocial behavior is associated positively with negative emotionality and daring and inversely with prosociality (Lahey & Waldman, 2003), but as detailed above, CADS dimensions have been found to be concurrently and predictively associated with a broad range of psychopathology.

### 1.2 Wave 2 sample and measures

Twin pairs for Wave 2 assessments were recruited in four replicates in reverse order of their age in Wave 1 (16–17, 14–15, 12–13, and 10–11 years) to minimize the age distribution in Wave 2. Twin pairs were eligible if the last known address of both twins was within 300 miles of Vanderbilt University (95.2% of twins). Wave 2 replicates were selected by oversampling on Wave 1 psychopathology scores based on the greater rating of each symptom from the parent or youth. High-risk pairs were selected with certainty if either twin had symptom ratings on the total number of internalizing, ADHD, or the combination of ODD and CD symptoms in the top 10% of that age range. In addition, 19–23% of the remainder of each replicate was randomly selected with two constraints: (1) monozygotic pairs were oversampled by randomly excluding 40% of the randomly selected dizygotic pairs and (2) the number selected from the remainder of the sample varied slightly to equate replicate sizes (100–105 pairs). Three pairs of twins could not be located, and 37 pairs refused screening. Eighteen selected pairs of twins across replicates were declared out of scope due to previous participation in a pilot study, mental or physical incapacity, residence outside the U.S., imprisonment, or death. A total of 114 screened individual twins were ineligible for neuroimaging based on feasibility (e.g., body weight) and safety reasons, but were eligible for assessment of psychopathology.

Assessments of adult psychopathology in Wave 2 were conducted in person at Vanderbilt University before neuroimaging or by telephone for scan-ineligible participants. Diagnostic and Statistical Manual, Fifth Edition (DSM5) symptoms were assessed using the young adult version of the Diagnostic Interview for Children (YA-DISC) (Abram et al., 2015; Shaffer, Fisher, Lucas, Dulcan, & Schwab-Stone, 2000; Witkiewitz et al., 2013). The modules used in these analyses queried diagnostic criteria for adult ADHD, major depressive disorder (MDD), generalized anxiety disorder (GAD), post-traumatic stress disorder (PTSD), agoraphobia, panic attacks, social phobia, specific phobia, manic episodes, obsessive–compulsive disorder, APD, and nicotine, alcohol, and marijuana misuse during the last 12 months. Because few skip patterns are in the YA-DISC, the instrument yields measures of the number of symptoms of each dimension of psychopathology even if a participant does not meet full criteria for a DSM5 diagnosis. Nonetheless, questions about abuse and dependence were only administered to those reporting use of the substance. Similarly, questions regarding symptoms of PTSD in the past year were only administered to participants who reported a traumatic event that they thought about during the last year. All GAD symptoms were queried only if the participant reported the cardinal symptom of frequent worry for at least 6 months in a row during the last year and were asked in the context of “when you were worried.” Therefore, not all possible PTSD, GAD, and substance use symptoms could contribute to symptom counts. Although participants were asked about all symptoms of depression, contingent questions used to set a threshold for the presence of each symptom based on its frequency and duration were asked only for persons reporting the symptoms of dysphoria and anhedonia, which may have increased the prevalence of endorsed depression symptoms.

### 1.3 Diffusion-weighted imaging (DWI) acquisition

During Wave 2, imaging data were acquired on two identical 3 T Intera-Achiava Phillips MRI scanners using a 32-channel head coil. T1-weighted images were acquired with a 3-D Magnetization Prepared Rapid Acquisition Gradient Echo (MPRAGE) sequence (TE/TR/TI = 4.6/9.0/644(shortest) ms; SENSE = 2.0; echo train=131; scan time = 4 min 32 s; FOV: 256 × 256 × 170 mm, 1 mm isotropic resolution). For DWI, we used a 5 min 2 s multi-slice Stejskal-Tanner spin echo sequence with an echo planar imaging readout (TE/TR = 52/7750 ms, SENSE = 2.2, FOV: 240 × 240 mm, 2.5 mm isotropic, 50 slices, 2.5 mm slice thickness). This was acquired with one image without diffusion weighting (“b_0_”) and 32 diffusion-weighted images equally distributed over a hemisphere (*b* = 1000 s/mm^2^).

## 2. Data Analysis

### 2.1 DWI data preprocessing

The DWI data were preprocessed based on methods detailed by Lauzon and colleagues (2013). The fMRIB’s Linear Image Registration Tool (FLIRT) from version 5.0.6 of the Functional Magnetic Resonance Imaging of the Brain Software Library (FSL; www.fmrib.ox.ac.uk/fsl) was used to register DWIs to the B_0_ volume using an affine registration with 12 degrees of freedom for eddy current and motion correction, and then the Brain Extraction Tool (BET) was used to mask the B_0_ volume (Jenkinson, Bannister, Brady, & Smith, 2002; Smith, 2002). Next FSL was used to perform eddy current and motion corrections. Then the CAMINO software package was used to implement RESTORE robust tensor fitting, which reduces the influence of motion-related artifacts (Chang, Jones, & Pierpaoli, 2005; Cook et al., 2006). After preprocessing, the data were quality checked for motion, FA bias and standard deviation, and goodness of fit of the data to the diffusion model (Lauzon et al., 2013). Participants were excluded if they were an outlier on any quality assurance metric. Next Tract-Based Spatial Statistics (TBSS) was run using FSL, which produced skeletonized white matter images based on the procedures detailed in Smith and colleagues (2006). Participants’ FA images were first moved to standard space based on a nonlinear transformation to the FMRIB58_FA template. Images were then averaged to create a mean FA image, thinned in order to derive a skeletonized mean image, and thresholded at FA > .2. FA images were projected onto the mean skeleton, which produced a 4D file that was used for statistical analyses. Because FA is a sensitive, but nonspecific indicator of atypical white matter microstructure (Alexander, Lee, Lazar, & Field, 2007), AD and RD skeletonized images also were created. This was done by applying the nonlinear warp used to bring each FA image to the template and applying each participant’s projection vectors onto the mean skeleton.

We used the JHU ICBM-DTI white matter labels atlas (Mori, Wakana, Van Zijl, & Nagae-Poetscher, 2005) to create masks of the following major white matter tracts: corpus callosum (body, genu, and splenium), corona radiata (anterior, superior, and posterior), internal capsule, external capsule, cingulum, posterior thalamic radiation, uncinate fasciculus, fornix, superior fronto-occipital fasciculus (SFOF), SLF, and sagittal stratum. Tract masks were overlaid with the white matter skeleton mask generated from the present sample, and only overlapping voxels were included in the final masks. We extracted average FA, AD, and RD across these masks to get tract-specific metrics of white matter microstructure and across the entire white matter skeleton to produce global metrics. A total of 427 participants completed a diffusion-weighted imaging (DWI) scan with complete data, but three were excluded owing to incomplete scan coverage and 17 participants were excluded because of excessive movement. Three tests of potential sample bias due to the exclusion of participants with excessive movement conducted in SAS 9.4 SURVEYLOGISTIC and revealed no significant associations of exclusion versus participation with the participant’s sex, age, or the three parent-reported or youth-reported CADS dispositions. Eight of the 410 participants did not have valid measures of TICV due to movement during T1-weighted image collection and were not included in analyses controlling TICV.

### 2.2 Regression analyses

Multiple regression analyses were performed using SAS 9.4 PROC SURVEYREG to adjust standard errors to reflect stratification and the clustering of twins within twin pairs. All analyses used weights that jointly (a) accounted for the inverse of the probability of selection into Wave 2 based on the selection strategy and (b) adjusted for any biases due to nonresponse or missing data after quality control relative to the participant’s age in Wave 2, sex, family income, maternal education, and Wave 1 measures of psychopathology, dispositions, and working memory using lasso logistic regression. These weights allow valid parameter estimates when weighted back to the full Wave 1 TTS sample (Korn & Graubard, 1999). We corrected for multiple testing using a false discovery rate (FDR) of 5% applied to two-tailed tests (Benjamini & Hochberg, 1995) in each family of statistical tests. Disposition scores and white matter integrity measures were standardized to a mean of 0 and standard deviation of 1 prior to analyses.

### 2.3 Preliminary tests of mean sex differences in white matter microstructure and behavior

Sex differences in weighted mean FA, AD, and RD averaged across the entire white matter skeleton, controlling for age in Wave 2, race-ethnicity, handedness, and scanner, were estimated, with and without the covariate of TICV. Similarly, sex differences in weighted mean measures of the CADS dispositions and the latent psychopathology dimensions were tested controlling for age in Wave 2 and race-ethnicity. These analyses took stratification and clustering within twin pairs into account in Mplus for latent variables and SAS 9.4 for measured variables.

### 2.4 Primary analyses of correlates of global fractional anisotropy

In separate regression models for each informant on the CADS, FA averaged across all skeletonized tracts was simultaneously regressed on the three CADS scales of negative emotionality, daring and prosociality, along with the demographic covariates of no interest (sex, age in Wave 1, age in Wave 2, and race-ethnicity). These analyses controlled for TICV, both because TICV is smaller on average in females (Gur & Gur, 2017; Ritchie et al., 2018) and there is some evidence that observed sex differences in FA could be an artifact of sex differences in TICV (Takao, Hayashi, & Ohtomo, 2014). Sensitivity analyses also were conducted without TICV as a covariate because controlling TICV is not a settled issue.

In a separate family of tests, interactions of sex with each CADS disposition in their associations with global FA were tested. Following sex-by-disposition interactions that were significant after FDR correction, post hoc regression analyses were conducted within each sex to facilitate interpretation of interactions.

### 2.5 Secondary analyses of tract-specific fractional anisotropy

In separate models for each of 15 individual skeletonized tracts, average FA of the tract was regressed on the three CADS scales of negative emotionality, daring and prosociality, along with the same demographic covariates of no interest as in the primary analyses.

### 2.6 Cingulum asymmetry

We attempted to replicate previous findings of an association between neuroticism and left/right asymmetry in FA of the cingulum (Madsen et al., 2012; Madsen, Jernigan, Vestergaard, Mortensen, & Baare, 2018) using the formula: Asymmetry =((2 * (Left – Right))/(Left + Right) * 100).

### 2.7 Exploratory tests of moderated mediation

In a frankly exploratory spirit, we tested for sex-moderated mediation by FA in each tract and averaged across all tracts of associations between dispositions and psychopathology factors defined in both bifactor and correlated factors models (Lahey, Zald et al., 2018) using multiple groups designs in Mplus. We conducted these tests only when there was a plausible basis for mediation based on previous findings using this sample and the current results. Specifically, mediation analyses were performed only when the following conditions were all met in the present sample: (i) CADS disposition *A* significantly predicted latent psychopathology factor *B* (Class et al., 2019); (ii) CADS disposition *A* significantly predicted FA in brain region *C*; and (iii) FA in brain region *C* was significantly associated with latent psychopathology factor *B*.

## 3. Results

Demographic characteristics of the 410 participants are in Supplemental Table S1. For supplementary material accompanying this paper, visit cambridge.org/PEN. Table S1 also presents the prevalence of 18 DSM-IV categorical mental disorders according to YA-DISC algorithms (Shaffer, Fisher, Piacentini, & Lucas, 2008) by sex. Consistent with oversampling, 50.2% met criteria for at least one Wave 2 mental disorder (44.2% of females; 55.8% of males) in the past year and 27.1% met criteria for ≥ 2 diagnoses. Brief versions of the items scored on each dimension in the parent- and youth-rated versions of the CADS are presented in Table S2 that shows the CADS dispositions were modestly correlated across raters.

### 3.1 Preliminary tests of sex differences in DWI and behavioral measures

After FDR adjustment for multiple statistical tests, the preliminary tests of mean sex differences in DWI-based measures (Table S3) revealed significant differences between females and males in TICV and in both global FA and RD averaged across all skeletonized white-matter tracts when age, race-ethnicity, handedness, scanner were controlled, whether TICV was controlled in the latter analyses. No sex differences in average AD were observed. Consistent with previous findings in a different sample (Lahey, Applegate et al., 2008; Lahey et al., 2010), there were no sex differences in parent-rated CADS negative emotionality, but males received higher ratings on average for parent-rated daring and lower ratings on parent-rated prosociality. In addition, females rated themselves as significantly higher on prosociality. When latent factors of adult psychopathology were defined in a correlated factors model, males had significantly higher scores on externalizing psychopathology. When latent factors of adult psychopathology were defined in a bifactor model, males had significantly higher scores on specific externalizing and lower scores on specific internalizing psychopathology.

### 3.2 Primary tests of associations with global measures of white matter integrity

**Figure 1.**
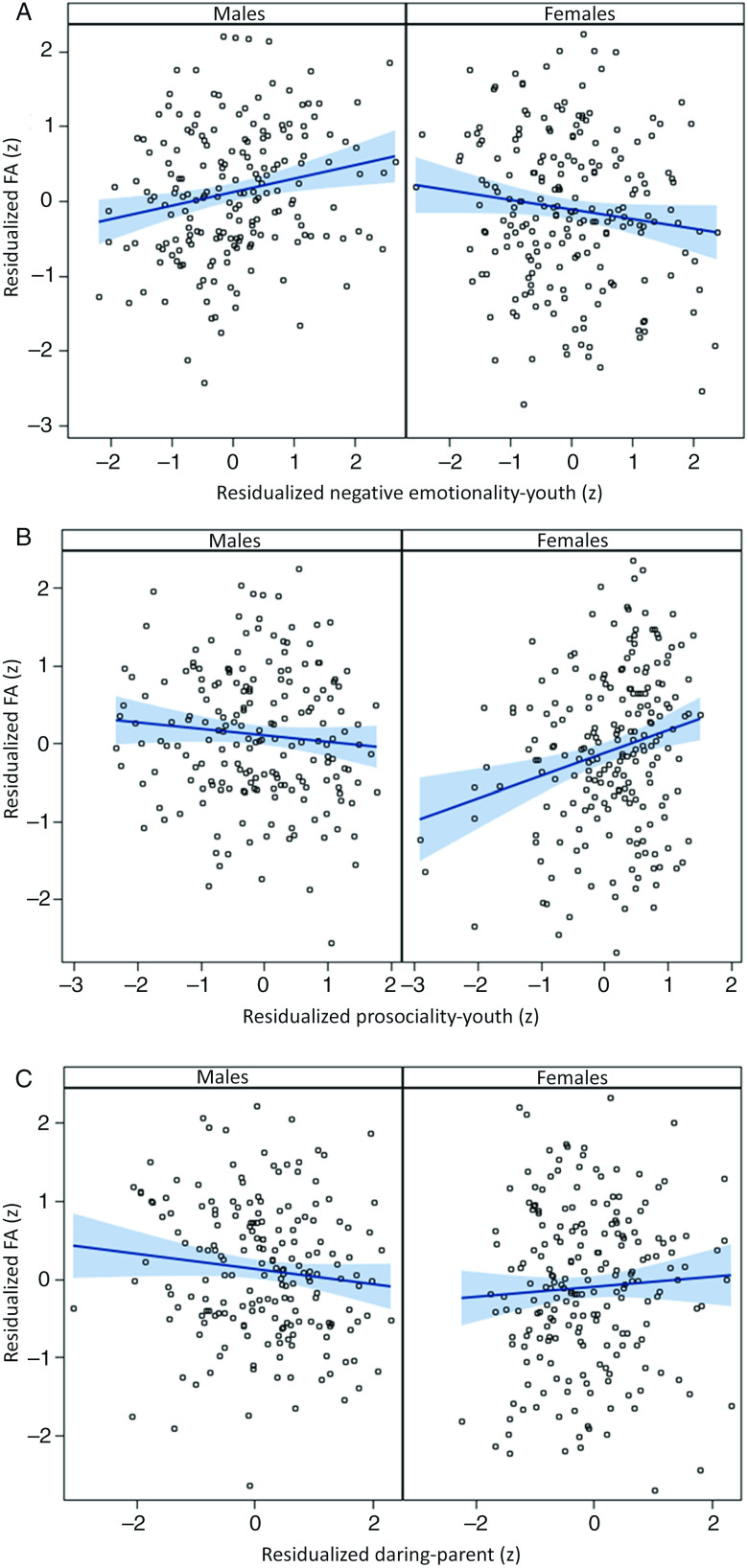
Residual–residual plots of sex-by-disposition interactions for (A) negative emotionality, (B) prosociality, and (C) daring rated at 10–17 years in predictive associations with mean fractional anisotropy across 12 skeletonized tracts in males and females at 22–31 years of age, with 95% confidence intervals for regression lines in blue. Variables on each axis are residualized on age in Wave 1, age in Wave 2, race-ethnicity, handedness, scanner, TICV, and the other two disposition scores.

The results in the top panels of Table 1 show that no CADS dimension was significantly associated after FDR correction with global FA averaged across the 15 tracts, whether TICV was controlled or not, although there were nominally significant associations of both parent- and youth-rated prosociality with global FA. In marked contrast, the bottom panels of Table 1 show significant interactions of sex with each of the three CADS dispositions in their associations with FA for one of the two raters, each of which survived FDR correction. As shown in Figure 1A, post hoc tests controlling TICV reported in Table 2 show that these significant interactions reflect a significant positive association between youth-rated negative emotionality and global FA in males, but a nominally significant inverse association in females. In addition, parent-rated prosociality was significantly related to global FA in females, but not males (Figure 1B), and parent-rated daring was significantly related to FA in males, but not in females (Figure 1C). Follow-up analyses (Tables S4 and S5) reveal that significant interactions with sex were also found for youth-rated negative emotionality and parent-rated daring for RD after FDR correction, but no associations or sex-by-disposition tests were significant for AD at corrected levels.

**Table 1.** Results of separate analyses in which the average of fractional anisotropy averaged over all tracts measured at 22–31 years of age was regressed simultaneously on CADS ratings of dispositions of negative emotionality, prosociality, and daring at 10–17 years of age and tests of sex-by-disposition interactions including covariates of no interest that did (upper rows) and did (lower rows) control total intracranial volume

Informant:	Parent		Youth	
Dispositions	β (SE)	P <	B (SE)	P <
Tests of Associations of Dispositions with Whole-Skeleton FA				
Covariates: Sex, age in Wave 1, age in Wave 2, race-ethnicity, handedness, scanner				
Negative emotionality	−0.06 (0.08)	0.4597	−0.05 (0.07)	0.4498
Prosociality	0.17 (0.08)	0.0235	0.18 (0.07)	0.0162
Daring	−0.08 (0.06)	0.1749	−0.03 (0.07)	0.6176
Covariates: Sex, age in Wave 1, age in Wave 2, race-ethnicity, handedness, scanner, TICV				
Negative emotionality	−0.05 (0.08)	0.5009	−0.04 (0.07)	0.5503
Prosociality	0.17 (0.08)	0.0234	0.18 (0.07)	0.0109
Daring	−0.08 (0.06)	0.1837	−0.03 (0.07)	0.6207
Tests of Sex-by-Disposition Interactions with Whole-Skeleton FA				
Covariates: Sex, age in Wave 1, age in Wave 2, race-ethnicity, handedness, scanner				
Negative emotionality	0.20 (0.15)	0.1885	−**0.31 (0.10)**	**0.0018**
Prosociality	**0.39 (0.17)**	**0.0240**	−0.07 (0.15)	0.6546
Daring	**0.28 (0.11)**	**0.0122**	0.14 (0.14)	0.2856
Covariates: Age in Wave 1, age in Wave 2, race-ethnicity, handedness, scanner, TICV				
Negative emotionality	0.21 (0.15)	0.1817	−**0.36 (0.10)**	**0.0004**
Prosociality	**0.40 (0.17)**	**0.0212**	−0.08 (0.15)	0.5617
Daring	**0.29 (0.11)**	**0.0103**	0.15 (0.13)	0.2554

CADS = Child and Adolescent Dispositions Scale; FA = fractional anisotropy; TICV = total intracranial volume.

CADS disposition scores standardized to mean of 0 and standard deviation of 1.

Coefficients in bold are significant after FDR correction (adopting a 5% false discovery rate) for 12 tests in a family of analyses of associations and a separate family of 12 tests of interactions with sex.

**Table 2.** Results of post hoc sex-stratified analyses in which whole-skeleton fractional anisotropy was regressed simultaneously on the three CADS dispositions and covariates of no interest in only males and only females to interpret sex-by-disposition interactions that were significant at FDR corrected levels (Table 1)

Informant:	Parent		Youth	
Dispositions	β (SE)	P <	B (SE)	P <
Males Only (N = 192)				
Negative emotionality	−0.17 (0.08)	0.0336	**0.18 (0.06)**	**0.0040**
Prosociality	−0.06 (0.08)	0.4768	0.18 (0.10)	0.0738
Daring	−**0.24 (0.07)**	**0.0016**	−0.13 (0.07)	0.0865
Females Only (N = 210)				
Negative emotionality	0.09 (0.12)	0.4669	−0.17 (0.08)	0.0273
Prosociality	**0.43 (0.12)**	**0.0008**	0.12 (0.10)	0.2216
Daring	0.06 (0.09)	0.4558	0.01 (0.10)	0.8828

CADS = Child and Adolescent Dispositions Scale. Covariates: age in Wave 1, age in Wave 2, race-ethnicity, handedness, scanner, total intracranial volume.

CADS disposition scores standardized to mean of 0 and standard deviation of 1.

Coefficients in bold are significant after FDR correction (adopting a 5% false discovery rate) for 12 tests.

### 3.3 Secondary tests of associations with measures of white matter integrity in individual tracts

The results of secondary tests of associations between CADS dispositions and FA in each of 15 individual skeletonized white matter tracts are presented in Table 3. After FDR correction, one association was significant: greater youth-rated prosociality significantly predicted greater FA in the genu of the corpus callosum. Furthermore, tests of sex-by-disposition interactions for these 15 individual tracts (Table 4) indicated that youth-rated negative emotionality predicted FA in the corpus callosum body, posterior corona radiata, and cingulum differentially in females and males after FDR correction. Similarly, there were significant sex differences in the associations of parent-rated daring with FA in the superior corona radiata and parent-rated prosociality with FA in the SFOF after FDR correction.

**Table 3.** Results of secondary tests of associations of fractional anisotropy in 15 individual skeletonized white matter tracts measured at 22–31 years of age on CADS ratings of negative emotionality, prosociality, and daring at 10–17 years of age controlling the other dispositions and demographic covariates of no interest,^a^ separately by informant on the dispositions (N = 410).

CADS informant:		Parent		Youth	
Outcome	Predictor	β (SE)	P <	B (SE)	P <
Corpus callosum (body)	NE	0.10 (0.09)	0.2585	−0.05 (0.11)	0.6592
	Prosociality	0.14 (0.08)	0.0943	0.10 (0.07)	0.1651
	Daring	−0.07 (0.07)	0.3214	−0.04 (0.07)	0.5835
Corpus callosum (genu)	NE	−0.04 (0.10)	0.6661	0.04 (0.06)	0.5181
	Prosociality	0.17 (0.09)	0.0713	**0.25 (0.06)**	**0.0001**
	Daring	0.02 (0.08)	0.7839	−0.02 (0.06)	0.6765
Corpus callosum (splenium)	NE	−0.15 (0.08)	0.0747	−0.08 (0.07)	0.2202
	Prosociality	−0.01 (0.07)	0.8593	0.12 (0.08)	0.1077
	Daring	−0.13 (0.05)	0.0107	−0.03 (0.08)	0.6835
Anterior corona radiata	NE	−0.10 (0.08)	0.2111	−0.01 (0.05)	0.7549
	Prosociality	0.25 (0.09)	0.0049	0.29 (0.09)	0.0013
	Daring	−0.03 (0.07)	0.7086	−0.10 (0.07)	0.2173
Superior corona radiata	NE	0.04 (0.08)	0.6098	0.03 (0.06)	0.5937
	Prosociality	0.17 (0.07)	0.0222	0.09 (0.07)	0.1938
	Daring	−0.08 (0.05)	0.1198	−0.07 (0.06)	0.2587
Posterior corona radiata	NE	−0.01 (0.07)	0.920	−0.00 (0.08)	0.9790
	Prosociality	0.06 (0.07)	0.403	0.06 (0.06)	0.3477
	Daring	−0.08 (0.07)	0.2435	−0.02 (0.07)	0.7672
Internal capsule	NE	−0.14 (0.07)	0.0480	−0.08 (0.04)	0.0958
	Prosociality	0.15 (0.07)	0.0451	0.09 (0.06)	0.1010
	Daring	0.01 (0.06)	0.8921	−0.05 (0.06)	0.3863
External capsule	NE	0.00 (0.08)	0.9861	−0.10 (0.12)	0.3865
	Prosociality	0.10 (0.06)	0.1091	0.17 (0.06)	0.0108
	Daring	−0.14 (0.09)	0.1372	0.10 (0.10)	0.2812
Cingulum	NE	−0.06 (0.07)	0.4329	−0.03 (0.04)	0.5476
	Prosociality	0.09 (0.07)	0.2109	0.12 (0.08)	0.1243
	Daring	−0.02 (0.07)	0.7582	−0.11 (0.06)	0.0918
Posterior thalamic radiation	NE	−0.13 (0.11)	0.2355	−0.06 (0.11)	0.6220
	Prosociality	0.02 (0.07)	0.7574	0.16 (0.09)	0.0877
	Daring	−0.15 (0.07)	0.0347	−0.01 (0.11)	0.9131
Uncinate fasciculus	NE	−0.01 (0.06)	0.8617	0.02 (0.07)	0.8139
	Prosociality	−0.02 (0.07)	0.7217	0.08 (0.08)	0.3130
	Daring	−0.12 (0.07)	0.0965	0.04 (0.08)	0.5525
Fornix	NE	0.00 (0.06)	0.9934	−0.05 (0.05)	0.3204
	Prosociality	0.17 (0.09)	0.0575	0.10 (0.08)	0.1905
	Daring	0.10 (0.07)	0.1397	0.02 (0.07)	0.6991
Superior Fronto-Occ Fasciculus	NE	−0.09 (0.08)	0.2628	−0.08 (0.06)	0.1692
	Prosociality	0.14 (0.08)	0.1032	0.13 (0.09)	0.1601
	Daring	−0.04 (0.07)	0.5837	−0.12 (0.07)	0.0973
Superior longitudinal fasciculus	NE	−0.08 (0.07)	0.2533	0.03 (0.05)	0.5404
	Prosociality	0.09 (0.07)	0.2335	0.13 (0.08)	0.1008
	Daring	−0.11 (0.07)	0.0897	−0.03 (0.07)	0.6765
Sagittal stratum	NE	−0.09 (0.10)	0.3936	−0.09 (0.14)	0.5084
	Prosociality	0.17 (0.07)	0.0164	0.23 (0.07)	0.0028
	Daring	−0.16 (0.06)	0.0124	0.00 (0.10)	0.9896

a Covariates of no interest: Age in Wave 1, age in Wave 2, sex, parent-classified race-ethnicity (Non-Hispanic white versus others), handedness scanner, and total intracranial volume.

Note: NE = negative emotionality; Occ = occipital; CADS scores and white matter tracts converted to z-scores;

Bold indicates significant after false discovery rate correction for 90 main effect tests for specific tracts.

**Table 4.** Results of secondary tests of *sex-by-disposition interactions* in regressions of fractional anisotropy in 15 separate skeletonized white matter tracts at 22–31 years of age on ratings of negative emotionality, prosociality, and daring measured at 10–17 years of age controlling the other dispositions and demographic covariates of no interest,^a^ separately by informant on the dispositions (N = 410)

CADS informant:		Parent		Youth	
Outcome	β (SE)	P <	B (SE)	P <	β (SE)
Corpus callosum (body)	NE	0.14 (0.19)	0.4663	−**0.53 (0.14)**	**0.0002**
	Prosociality	0.33 (0.17)	0.0584	0.02 (0.15)	0.8902
	Daring	0.17 (0.12)	0.1584	−0.06 (0.13)	0.6583
Corpus callosum (genu)	NE	0.24 (0.17)	0.1540	−0.29 (0.10)	0.0041
	Prosociality	0.28 (0.20)	0.1770	−0.24 (0.12)	0.0475
	Daring	0.21 (0.14)	0.1349	0.03 (0.13)	0.7989
Corpus callosum (splenium)	NE	0.27 (0.13)	0.0397	−0.22 (0.12)	0.0658
	Prosociality	0.12 (0.14)	0.3893	−0.04 (0.14)	0.7718
	Daring	0.05 (0.12)	0.6721	0.10 (0.12)	0.3969
Anterior corona radiata	NE	0.18 (0.14)	0.1853	−0.07 (0.10)	0.4813
	Prosociality	0.23 (0.17)	0.1804	−0.13 (0.13)	0.3417
	Daring	0.19 (0.12)	0.1365	0.16 (0.16)	0.3370
Superior corona radiata	NE	0.11 (0.14)	0.4002	−0.27 (0.11)	0.0173
	Prosociality	0.35 (0.17)	0.0372	−0.17 (0.14)	0.2194
	Daring	**0.31 (0.10)**	**0.0013**	0.21 (0.11)	0.0655
Posterior corona radiata	NE	−0.04 (0.14)	0.7881	−**0.40 (0.11)**	**0.0003**
	Prosociality	0.35 (0.15)	0.0220	−0.17 (0.12)	0.1648
	Daring	0.29 (0.14)	0.0339	0.18 (0.10)	0.0833
Internal capsule	NE	0.13 (0.14)	0.3443	−0.11 (0.09)	0.2122
	Prosociality	0.32 (0.16)	0.0502	−0.06 (0.12)	0.5830
	Daring	0.20 (0.10)	0.0536	0.18 (0.13)	0.1608
External capsule	NE	0.16 (0.15)	0.2714	−0.42 (0.15)	0.0053
	Prosociality	0.22 (0.16)	0.1730	0.02 (0.14)	0.8735
	Daring	−0.04 (0.19)	0.8572	0.15 (0.14)	0.2985
Cingulum	NE	0.22 (0.14)	0.1139	−**0.26 (0.08)**	**0.0028**
	Prosociality	0.34 (0.17)	0.0473	−0.01 (0.15)	0.9600
	Daring	0.14 (0.12)	0.2475	0.13 (0.15)	0.3874
Posterior thalamic radiation	NE	0.22 (0.15)	0.1513	−0.26 (0.17)	0.1215
	Prosociality	0.09 (0.16)	0.5876	−0.24 (0.14)	0.0841
	Daring	0.07 (0.15)	0.6360	0.26 (0.13)	0.0451
Uncinate fasciculus	NE	0.01 (0.11)	0.9082	−0.14 (0.10)	0.1600
	Prosociality	0.01 (0.14)	0.9315	−0.04 (0.16)	0.8069
	Daring	0.11 (0.14)	0.4409	0.14 (0.13)	0.2564
Fornix	NE	0.16 (0.13)	0.2215	−0.13 (0.10)	0.1758
	Prosociality	0.11 (0.15)	0.4432	−0.03 (0.16)	0.8526
	Daring	0.03 (0.14)	0.8001	0.00 (0.14)	0.9741
Superior Fronto-Occ Fasciculus	NE	0.26 (0.15)	0.0939	−0.14 (0.11)	0.2120
	Prosociality	**0.49 (0.15)**	**0.0019**	0.03 (0.17)	0.8643
	Daring	0.20 (0.12)	0.0875	0.14 (0.14)	0.3183
Superior longitudinal fasciculus	NE	0.09 (0.12)	0.4290	−0.25 (0.10)	0.0124
	Prosociality	0.17 (0.14)	0.2044	−0.19 (0.15)	0.2094
	Daring	0.34 (0.12)	0.0065	0.36 (0.13)	0.0066
Sagittal stratum	NE	0.18 (0.17)	0.2851	−0.28 (0.20)	0.1643
	Prosociality	0.13 (0.16)	0.4291	−0.24 (0.12)	0.0466
	Daring	0.09 (0.13)	0.4868	0.14 (0.12)	0.2543

a Covariates of no interest: Age in Wave 1, age in Wave 2, parent-classified race-ethnicity (Non-Hispanic white versus others), handedness, scanner, and total intracranial volume.

Note: NE = negative emotionality; Occ = occipital; CADS scores and white matter tracts converted to z-scores.

Bold indicates significant after 5% false discovery rate correction for 90 tests of sex-by-disposition interactions for specific tracts.

### 3.4 Post hoc tests following sex-by-disposition interactions

Table S6 presents the results of sex-stratified tests of associations of each tract for which there was a significant sex-by-disposition (Table 4). Youth-rated negative emotionality was positively associated with the corpus callosum body, *β* = 0.30, *p* < 0.0012) and the posterior corona radiata, *β* = 0.22, *p* < 0.0068, after FDR correction in males only. The direction of association was in the opposite direction in females, but was not significant after FDR correction in the corpus callosum body or the posterior corona radiata. The directions of association with youth-rated negative emotionality and the cingulum were positive for males and negative for females, but neither association was significant after FDR correction. For parent-rated daring, the association with the superior corona radiata was significant for males only, *β* = −0.24, *p* < .0008. In contrast, among females only, the associations of parent-rated prosociality were significant after FDR correction with the superior corona radiata, *β* = 0.38, *p* < 0.0022, and the SFOF, *β* = 0.36, *p* < 0.0004.

### 3.5 Exploratory follow-up tests of significant sex-by-disposition interactions

For tracts with significant sex-by-disposition interactions after FDR correction (Table 4), follow-up tests were conducted for the relevant CADS informant for FA in each tract in the left and right hemispheres, except for the corpus callosum which connects the hemispheres. For the cingulum, these follow-up tests were conducted separately for left and right cingulate gyrus and parahippocampal segments to further localize the atypicality. As shown in Table S7, each significant interaction in Table 4 was significant bilaterally (i.e., separately in both left and right tracts). There were significant bilateral interactions with sex for the cingulum-cingulate gyrus segment, but no significant interactions for the parahippocampal segment.

### 3.6 Cingulum asymmetry

We failed to replicate the findings of Madsen et al. (2012, 2018). The ratio of left/right asymmetry in FA of the cingulum was not significantly associated with any CADS disposition at *p* < .05, and no sex-by-disposition interaction was significant.

### 3.7 Exploratory tests of sex-moderated mediation

Table S8 presents findings on the three conditions which must be met for a plausible test of mediation: (i) the disposition predicts psychopathology; (ii) the same disposition predicts FA in a tract; and (iii) FA in the same tract is associated with the same psychopathology dimension. These conditions were considered using both recently published results based on this sample (Class et al., 2019; Hinton et al., 2019) and new analyses of the present data, all of which are summarized in Table S8. Based on psychopathology dimensions defined in the correlated factors model, five plausible paths were identified through which regional FA might mediate the association of dispositions with latent psychopathology factors defined in a correlated factors model: (1) youth-rated negative emotionality through cingulum FA to externalizing psychopathology; (2) youth-rated negative emotionality through cingulum FA to internalizing psychopathology; (3) youth-rated negative emotionality through posterior corona radiata FA to externalizing psychopathology; (4) youth-rated prosociality through corpus callosum genu FA to externalizing; and (5) youth-rated negative emotionality through corpus callosum body FA to externalizing psychopathology. As shown in Figure S1, however, sex-moderated mediation was identified tentatively only for the latter path. The difference between females and males in the mediated path from youth-rated negative emotionality through corpus callosum body FA to externalizing psychopathology was significant, *z* = 2.89, *p* < 0.015. This reflected both a significant direct path between negative emotionality and externalizing, *β* = 0.24 (SE: 0.07), *p* < 0.001, and an indirect path through the corpus callosum body FA, *β* = 0.07 (SE: 0.03), *p* < 0.042, in males, but nonsignificant direct, *β* = 0.08 (0.08), *p* < 0.322, and indirect, *β* = −0.06 (0.04), *p* < 0.089, paths for females. No plausible mediated paths were identified for the latent general, specific externalizing, and specific internalizing defined in bifactor models.

## 4. Discussion

The present analyses confirmed previous reports of sex differences in FA in the brains of females and males population (Ritchie et al., 2018; Tamnes et al., 2018; van Hemmen et al., 2017) and revealed significant sex differences in predictive associations between CADS dispositional constructs rated in childhood and adolescence and FA across the white matter skeleton in early adulthood. Sex-stratified post hoc tests revealed that these interactions reflect associations in opposite directions between the disposition and global FA in females and males. The association with global FA was statistically significant after FDR correction in only one sex in each case. This could result from limited statistical power for tests among only one sex, but effect sizes were very small in the sex without a significant association. Follow-up tests identified similarly robust sex-by-disposition interactions for global RD but not AD which suggests a possible association between dispositions and individual differences in aspects of myelination. Critically, these findings suggest the need to carefully consider the sex of the participant in studies of the neural correlates of temperament and personality, as it is possible that at least some correlates are sex specific.

Secondary analyses of FA in each of the 15 white matter tracts separately showed that greater youth-rated prosociality predicted higher mean FA in the corpus callosum genu (*p* < .0001) after FDR correction in the absence of sex moderation (Tables 3 and 4). All other significant predictive associations between dispositions and FA in specific tracts were moderated by sex. These findings should focus future research on these tracts, but it is important to consider that the observed associations with specific tracts may partially reflect the sizes of the tracts. Because these sex-moderated associations were found mostly in large white matter tracts (cingulum bundle, genu, and corona radiata), they may simply have been detected more readily than interactions involving smaller tracts.

Nonetheless, follow-up tests indicated that the sex-by-disposition interactions were significant after FDR correction in the superior and posterior corona radiata and the SFOF in both left and right hemispheres and were found bilaterally in the cingulate gyrus segment of the cingulum bundle, each of which is potentially relevant to dispositions. The superior and posterior corona radiata connect subcortical structures with frontal and parietal cortical regions implicated in emotion regulation and executive functions (Stave et al., 2017), and lower FA in these tracts has been linked to mental disorders, including ADHD (Chuang, Wu, Huang, Weng, & Yang, 2013; Cortese et al., 2013; Nagel et al., 2011; Onnink et al., 2015) and depression (Benedetti et al., 2011; Ota et al., 2015). The cingulum is a major pathway of the limbic system, and a recent review suggests that lower FA in the cingulate portions of the cingulum is associated with deficient emotion regulation, executive control, and a broad range of psychopathology (Bubb, Metzler-Baddeley, & Aggleton, 2018). Greater youth-rated prosociality significantly predicted greater FA in the genu of the corpus callosum after FDR correction. The genu connects the left and right prefrontal cortices and deficits in white matter integrity in the genu have been inconsistently linked to callousness and psychopathy (Sethi et al., 2018). Finally, we found a sex-moderated association between prosociality and FA in the SFOF, which connects prefrontal, parietal, and occipital regions. This is interesting because a recent review reported an association between FA in the SFOF and lower empathy (Comes-Fayos, Romero-Martinez, & Moya-Albiol, 2018).

Although we observed significant associations between FA and CADS dispositions rated by one informant, these did not replicate using the other raters’ scoring. This is consistent with previous studies reporting only low to moderate agreement between informant ratings of temperament and personality traits across youth self-reports and adult raters (Boson, Brandstrom, & Sigvardsson, 2018; Capaldi & Rothbart, 1992; Quilty, Cosentino, & Bagby, 2018; Tackett, 2011). Similarly, CADS dimensions rated by parents and youth are only modestly correlated (Class et al., 2019). Such modest correlations between parent and youth ratings of dispositions may reflect differences between the raters in maturity, experiences, the situations in which they observe the child, the covertness of some important experiences, and response style differences between the informants.

## 5. Limitations

The present findings suggest that variations in child and adolescent dispositions may predict variations in white matter microstructure in adulthood, but often differently in females and males. The absence of concurrent assessments of dispositions and white matter microstructure at the same ages limits the interpretation of these findings. Because myelination is dynamic process that is influenced by experience (Thomason & Thompson, 2011), future research will need to determine if the observed variation in FA was concurrently correlated with dispositions during childhood and adolescence. If not, the correlations with dispositions because the dispositions are transactively linked to experiences that influence white matter integrity over time.

It may be noted that concerns have been raised that the eddy_correct method in version 5.0.6 of FLS does not reduce signal attenuation as much as newer methods (Graham, Drobnjak, & Zhang, 2016). Because the most likely effect of inadequate attenuation of noise is reduced sensitivity and statistical power, additional significant associations may have been detected had a newer version of FSL had been used. It is possible, nonetheless, that signal attenuation resulted in artefactual associations.

### 5.1 Implications for quantitatively derived dimensional models of psychopathology

Understanding the psychobiological substrates of psychopathology is made challenging by the multidetermined and transactional nature of psychopathology that includes both dispositional features that precede the development of overt psychopathology as well as more temporally proximal processes directly related to the expression of symptoms or their consequence. Indeed, in some cases, a focus on just current psychopathology may obscure associations. Consistent with the Research Domains (RDoC) hypothesis (Cuthbert & Insel, 2013) and many other theoretical statements (Barlow, Sauer-Zavala, Carl, Bullis, & Ellard, 2014; Clark, Watson, & Mineka, 1994; Eysenck, White, & Eysenck, 1976; Lahey et al., 2017), it is likely that psychopathology, be it broad latent factors of psychopathology or first-order dimensions, is heterogeneous in the sense of reflecting individual differences in varying combinations of multiple dispositional and psychological processes (Lahey et al., 2017). Because of their stability, and strong association with subsequent psychopathology, a focus on dispositional characteristics may provide an important leg up in elucidating the neural features of psychopathology. Therefore, it may be easier to identify individual differences in these biopsychological processes by studying the neural correlates of dispositional constructs in addition to psychopathology than studying only psychopathology.

A focus on dispositional contributions to psychopathology also may be useful in understanding heterogeneity of psychopathology. For example, all three CADS dispositional dimensions have been found to be correlated concurrently and predictively with the externalizing dimension of psychopathology (Lahey, Class et al., 2018; Mikolajewski et al., 2013b; Shaw et al., 2012; Sitnick, Brennan et al., 2014; Sitnick, Shaw et al., 2014; Trentacosta et al., 2009). This suggests that no single disposition or related biopsychological mechanism in isolation is likely to provide a completely satisfactory understanding of externalizing psychopathology. It seems reasonable to hypothesize that the same is true of other second-order latent factors of psychopathology (Lahey et al., 2017). Therefore, the simultaneous assessment of multiple dispositions may be required when incorporating dispositions into clinical neuroscience studies. The present findings suggest that the approach taken by the CADS to measuring individual differences in multiple dispositional domains may be among the useful approaches to discovering the neural correlates of dimensions of psychopathology.

The need to consider heterogeneity in psychobiological processes is further emphasized by the observed interactions with sex. That associations differed so dramatically based on sex suggest that the consideration of sex differences in brain–behavior relations must be front and center in future studies of psychobiological links between temperament and psychopathology. Other recent findings suggest that other potential moderators, such as adverse experiences and poverty, also must be considered (Deater-Deckard, Li, Lee, King-Casas, & Kim-Spoon, 2019).

The exploratory tests of moderated mediation of predictive associations between dispositions and psychopathology factors by individual differences in FA used a statistical strategy that may improve understanding of developmental relations between dispositions and psychopathology at biological levels of analysis in the future. We identified plausible paths for which such mediation may occur in the correlated factors model, although we did not find any significant mediation paths for the bi-factor model. The exploratory nature of analyses cannot be overemphasized, however. The three plausible mediated paths tested for mediation were selected after examining 480 tests of significance (5 psychopathology factors defined in bifactor and correlated factors models X 3 CADS dimensions X 2 CADS informants X 16 whole-brain and regional measures of FA), which raises the very real possibility that the significant tests of mediation were based on chance associations. Thus, the present finding of sex-moderated mediation certainly requires replication. Hopefully, future longitudinal studies will employ larger sample sizes and will use more frequent repeated measurements of dispositions, psychopathology, and individual differences in brain at multiple ages to address such issues of mediation.

## Supplementary material

For supplementary material accompanying this paper visit https://doi.org/10.1017/pen.2020.3. click here to view supplementary material
