# Supplementary material for: Sex differences in associations of socioemotional dispositions measured in childhood and adolescence with brain white matter microstructure 12 years later
Source: Personal Neurosci. 2020 May 13;3:e5. doi: 10.1017/pen.2020.3 (PMC7253690; doi:10.1017/pen.2020.3)
Supplement: Supplementary file 1 [file S2513988620000036sup001.docx]

Supplemental Tables and Figure

Socioemotional Dispositions in Childhood and Adolescence are Differentially Associated with

Brain White Matter Microstructure 12 Years Later in Females and Males

Benjamin B. Lahey, Kendra E. Hinton, Francisco Calvache Meyer, Victoria Vallalta-Gil, Paul J. Rathouz,

Brooks Applegate, Xiaochan Yang, and David H. Zald

Table S1. Demographic characteristics and the prevalence of DSM-IV mental disorders in the sample of 410^a^ with analyzable DWI data.

| Characteristic |  |
| --- | --- |
| Sex (% female) | 52.2 |
| Race-ethnic group (%) |  |
| Non-Hispanic white | 72.0 |
| African American | 24.9 |
| Other groups | 3.2 |
| Handedness (% right) | 90.5 |
| Family income^b^ | 18.8 (5.0) |
| Years of maternal education (mean, SD) | 13.6 (2.7) |
| Age in years (mean, SD) |  |
| Wave 1 | 13.6 (2.5) |
| Wave 2 | 26.0 (1.8) |
| Scanner (% 3TA)^c^ | 52.2 |
|  |  |

*Diagnoses* *Females Males*

N = 214 N = 196

*N (%) N (%)*

Specific phobia 23 (10.8) 13 (6.6)

Agoraphobia 16 (7.5) 9 (4.6)

Panic disorder 9 (4.2) 10 (5.1)

Post-traumatic stress disorder 8 (3.7) 4 (2.0)

Social anxiety disorder 18 (8.4) 14 (7.1)

Obsessive-compulsive disorder 12 (5.6) 11 (5.6)

Generalized anxiety disorder 10 (4.7) 7 (3.6)

Major depression 14 (6.5) 14 (7.1)

Dysthymia 0 (0.0) 0 (0.0)

Attention-deficit/hyperactivity disorder 5 (2.3) 10 (5.1)

Alcohol abuse 24 (11.2) 42 (21.5)

Alcohol dependence 6 (2.8) 12 (6.1)

Cannabis abuse 11 (5.1) 24 (12.2)

Cannabis dependence 5 (7.8) 11 (5.6)

Nicotine dependence 16 (7.8) 21 (11.6)

Antisocial personality disorder 14 (6.5) 42 (21.4)

Hypomania 3 (1.4) 5 (2.6)

Mania 0 (0.0) 0. (0.0)

^a^N = 402 when total intracranial volume was controlled.

^b^Family income from wave 1 reported in brackets ranging from 0 (no income) to 24 ($100,000 and over in the year 2000), where and ordinal ranking of 18 = $35,000 - $44,999;

^c^Imaging data were acquired on two identical 3T Intera-Achieva Phillips MRI scanners.

Table S2. Items scored on the three CADS disposition scales based on factor analyses and Spearman correlations among the dimensions in the subsample used in the present analyses (N = 499).

Parent Ratings Youth Ratings

## *Prosociality Scale*

Cares about others' feelings Cares about others' feelings

Concerned about others who are hurt Concerned about others who are hurt

Spontaneously helps others Spontaneously helps others

Cheers up others when sad Cheers up others when sad

Feels sorry for kids who get picked on Feels sorry for kids who get picked on

Would be upset if saw animal get hurt Would be upset if saw animal get hurt

Concerned about right and wrong Concerned about right and wrong

Wants everyone to follow the rules Wants everyone to follow the rules

Would feel guilty if broke a law Would feel guilty if broke a law

Tries to do excellent work Likes meeting people

Enjoys learning interesting things Friendly

Spontaneously shares Enjoys being with others

Enjoys being praised

## *Negative Emotionality Scale*

Gets upset easily Gets upset easily

Reacts intensely Reacts intensely

Moods change unpredictably Moods change unpredictably

Blows things out of proportion Blows things out of proportion

Jealous Jealous

Gets bored easily Gets bored easily

Easily embarrassed Easily embarrassed

Emotional

Calm and easy-going (inverse)

## *Daring Scale*

Daring and adventurous Daring and adventurous

Enjoys risky and dangerous things Enjoys risky and dangerous things

Likes rough games and sports Likes rough games and sports

Likes things that are exciting and loud Likes things that are exciting and loud

Brave Brave

|  | Parent Informant | | | Youth Informant | | |
| --- | --- | --- | --- | --- | --- | --- |
|  | Negative emotionality | Prosociality | Daring | Negative emotionality | Prosociality | Daring |
| Parent Informant: |  | | | | | |
| Negative emotionality |  |  |  |  |  |  |
| Prosociality | -0.30*** |  |  |  |  |  |
| Daring | 0.21*** | -0.23*** |  |  |  |  |
| Youth Informant: |  | | | | | |
| Negative emotionality | *0.25**** | -0.14** | -0.01 |  |  |  |
| Prosociality | -0.11* | *0.39**** | -0.17*** | -0.06 |  |  |
| Daring | 0.07 | -0.18*** | *0.41**** | 0.03 | -0.08 |  |

Italics indicate cross-informant correlations of ratings of the same dispositions.

* p < .05; ** p < .01; *** p < .001

Table S3. Results of tests of sex differences in weighted standardized mean fractional anisotropy, radial diffusivity, and axial diffusivity averaged across all 15 skeletonized white matter tracts, weighted standardized mean CADS disposition scores by informant, and weighted standardized latent dimensions of psychopathology defined in both correlated factors and bifactor models.

|  | Sex-stratified Means | | Tests of Sex Difference | | | |
| --- | --- | --- | --- | --- | --- | --- |
| Measures | Females | Males |  |  |  |  |
|  | Mean (SE) | Mean (SE) | Estimate (SE)^1^ | P < | Estimate (SE)^2^ | P |
| *Brain Measures* | N = 210 | N = 192 |  |  |  |  |
| Total intracranial volume (z-scores) | -0.52 (0.11) | 0.62 (0.11) | **-1.10 (0.12)** | **0.0001** |  |  |
| *DWI indices* | N = 214 | N = 196 |  | | | |
| Fractional anisotropy (whole skeleton z-scores) | -0.23 (0.18) | 0.27 (0.08) | **-0.44 (0.15)** | **0.0030** | **-0.39 (0.17)** | **0.0244** |
| Radial diffusivity (whole skeleton z-scores) | 0.13 (0.14) | -0.18 (0.08) | **0.30 (0.13)** | **0.0205** | **0.47 (0.16)** | **0.0036** |
| Axial diffusivity (whole skeleton z-scores) | -0.12 (0.09) | 0.10 (0.09) | -0.16 (0.12) | 0.2028 | 0.21 (0.15) | 0.1744 |
| *Behavioral Measures* | N = 214 | N = 196 | Estimate (SE)^3^ | P < |  |  |
| *CADS Dimension Scores (Parent-rated)* | | | | | | |
| Negative emotionality | -0.34 (0.08) | -0.26 0.07) | -0.06 ( 0.14) | 0.6389 |  |  |
| Prosociality | 0.32 (0.12) | -0.03 (0.08) | **0.39 (0.11)** | **0.0008** |  |  |
| Daring | -0.31 (0.10) | 0.09 (0.10) | **-0.38 (0.14)** | **0.0052** |  |  |
| *CADS Dimension Scores (Youth-rated)* | | | | | | |
| Negative emotionality | -0.18 ( 0.09) | -0.20 (0.10) | 0.01 (0.14) | 0.9414 |  |  |
| Prosociality | 0.42 (0.07) | -0.20 (0.10) | **0.60 (0.14)** | **0.0001** |  |  |
| Daring | -0.16 (0.10) | 0.05 (0.10) | -0.21 (0.14) | 0.1430 |  |  |
| *Psychopathology Dimensions (Correlated Factors Model)*^4^ | | | | | | |
| Internalizing | -0.08 (0.09) | -0.36 (0.06) | 0.13 (0.07) | 0.074 |  |  |
| Externalizing | 0.26 (0.08) | 0.84 (0.08) | **-0.27 (0.06)** | **0.001** |  |  |
| *Psychopathology Dimensions (Bifactor Model)*^4^ | | | | | | |
| General | 0.11 (0.08) | -0.15 (0.08) | -0.13 (0.07) | 0.058 |  |  |
| Specific internalizing | 0.26 (0.11) | -0.22 (0.06) | **0.22 (0.08)** | **0.009** |  |  |
| Specific externalizing | 0.23 (0.08) | 0.78 (0.09) | **-0.27 (0.09)** | **0.002** |  |  |

^1^Controlling age in wave 2, race-ethnicity, handedness, and scanner (N= 410) .

^2^Controlling age in wave 2, race-ethnicity, handedness, scanner, and total intracranial volume (N = 402).

^3^Controlling age in wave 2 and race-ethnicity.

^4^Means of estimated standardized factor scores; tests of sex difference based on latent factor scores.

Sex coded: female = 1, male = 0; DWI = diffusion-weighted imaging.

Regression coefficients in bold are significant after 5% false discovery rate correction for 18 statistical tests.

Table S4. Results of follow-up analyses in which whole-skeleton radial diffusivity was regressed simultaneously on the three CADS dispositions and tests of sex-by-disposition interactions including covariates of no interest that did (upper rows) and did (lower rows) control total intracranial volume.

| Informant: | Parent |  | Youth |  |
| --- | --- | --- | --- | --- |
| Dispositions | β (SE) | P < | Β (SE) | P < |
| Tests of Associations of Dispositions with Whole-Skeleton RD | | | | |
| Covariates: Sex, age in wave 1, age in wave 2, race-ethnicity, handedness, scanner | | | | |
| Negative emotionality | 0.04 (0.08) | 0.6549 | 0.02 (0.07) | 0.8090 |
| Prosociality | -0.11 (0.08) | 0.1499 | -0.19 (0.08) | 0.0120 |
| Daring | 0.08 (0.06) | 0.2195 | -0.03 (0.06) | 0.6612 |
| Covariates: Sex, age in wave 1, age in wave 2, race-ethnicity, handedness, scanner, TICV | | | | |
| Negative emotionality | 0.05 (0.08) | 0.5017 | 0.02 (0.07) | 0.7260 |
| Prosociality | -0.14 (0.08) | 0.0865 | -0.18 (0.08) | 0.0145 |
| Daring | 0.08 (0.06) | 0.2048 | -0.01 (0.06) | 0.8187 |
| Tests of Sex-by-Disposition Interactions with Whole-Skeleton RD | | | | |
| Covariates: Sex, age in wave 1, age in wave 2, race-ethnicity, handedness, scanner | | | | |
| Negative emotionality | -.13 (0.15) | 0.3717 | **0.28 (0.10)** | **0.0043** |
| Prosociality | -0.22 (0.16) | 0.1700 | 0.18 (0.14) | 0.2152 |
| Daring | -0.27 (0.12) | 0.0251 | -0.08 (0.12) | 0.4853 |
| Covariates: Sex, age in wave 1, age in wave 2, race-ethnicity, handedness, scanner, TICV | | | | |
| Negative emotionality | -0.12 (0.15) | 0.4168 | **0.28 (0.10)** | **0.0056** |
| Prosociality | -0.21 (0.16) | 0.1979 | 0.18 (0.14) | 0.2124 |
| Daring | **-0.30 (0.12)** | **0.0124** | -0.10 (0.12) | 0.4397 |

CADS = Child and Adolescent Dispositions Scale; RD = radial diffusivity; TICV = total intracranial volume.

Coefficients in bold are significant after FDR correction for 12 tests in each family of analyses with and without control for total intracranial volume (adopting a 5% false discovery rate).

AAAA LOCKED SURVEYREG TBSS 11 JULY 2018

Table S5. Results of follow-up analyses in which whole-skeleton axial diffusivity was regressed simultaneously on the three CADS dispositions and tests of sex-by-disposition interactions including covariates of no interest that did (upper rows) and did (lower rows) control total intracranial volume.

| Informant: | Parent |  | Youth |  |
| --- | --- | --- | --- | --- |
| Dispositions | β (SE) | P < | Β (SE) | P < |
| Tests of Associations of Dispositions with Whole-Skeleton AD | | | | |
| Covariates: Sex, age in wave 1, age in wave 2, race-ethnicity, handedness, scanner | | | | |
| Negative emotionality | -0.02 (0.07) | 0.7598 | -0.04 (0.06) | 0.5106 |
| Prosociality | 0.04 (0.06) | 0.5511 | -0.08 (0.06) | 0.2360 |
| Daring | 0.02 (0.06) | 0.6987 | -0.09 (0.05) | 0.0792 |
| Covariates: Sex, age in wave 1, age in wave 2, race-ethnicity, handedness, scanner, TICV | | | | |
| Negative emotionality | 0.02 (0.06) | 0.8153 | -0.01 (0.06) | 0.8790 |
| Prosociality | -0.00 (0.06) | 0.9547 | -0.06 (0.06) | 0.3533 |
| Daring | 0.03 (0.06) | 0.6248 | -0.07 (0.05) | 0.1822 |
| Tests of Sex-by-Disposition Interactions with Whole-Skeleton AD | | | | |
| Covariates: Sex, age in wave 1, age in wave 2, race-ethnicity, handedness, scanner | | | | |
| Negative emotionality | 0.08 (0.13) | 0.5188 | 0.08 (0.11) | 0.4314 |
| Prosociality | 0.14 (0.11) | 0.1759 | 0.26 (0.10) | 0.0128 |
| Daring | -0.09 (0.13) | 0.4688 | 0.09 (0.10) | 0.3603 |
| Covariates: Sex, age in wave 1, age in wave 2, race-ethnicity, handedness, scanner, TICV | | | | |
| Negative emotionality | 0.12 (0.12) | 0.3201 | 0.01 (0.11) | 0.8936 |
| Prosociality | 0.17 (0.11) | 0.1212 | 0.24 (0.11) | 0.0238 |
| Daring | -0.14 (0.13) | 0.2863 | 0.08 (0.10) | 0.4280 |

CADS = Child and Adolescent Dispositions Scale; AD = axial diffusivity; TICV = total intracranial volume.

No coefficients were significant after FDR correction for 12 tests in each family of analyses with and without control for total intracranial volume (adopting a 5% false discovery rate).

AAAA LOCKED SURVEYREG TBSS 11 JULY 2018

Table S6. Results of post hoc sex-stratified regressions conducted following significant sex-by-disposition interactions shown in Table 4 in their associations with fractional anisotropy, controlling the other dispositions and demographic covariates of no interest,^a^ separately by informant on the dispositions, separately by sex and by informant on the dispositions.

|  |  |
| --- | --- |

| CADS informant: | | Parent | | | | Youth | | | |
| --- | --- | --- | --- | --- | --- | --- | --- | --- | --- |
| Sex of Participant: | | Males (n = 196) | | Females (n = 214) | | Males (n = 196) | | Females (n = 214) | |
| Outcome | CADS Predictor | β (SE) | P < | β (SE) | P < | β (SE) | P < | β (SE) | P < |
| Corpus callosum (body) | NE^1^ |  |  |  |  | **0.30 (0.09)** | **0.0012** | -0.26 (0.11) | 0.0177 |
|  | Prosociality |  |  |  |  | 0.07 (0.10) | 0.4900 | 0.08 (0.09) | 0.4196 |
|  | Daring |  |  |  |  | -0.03 (0.07) | 0.6724 | -0.11 (0.09) | 0.2434 |
| Posterior corona radiata | NE^1^ |  |  |  |  | **0.22 (0.08)** | **0.0068** | -0.15 (0.08) | 0.0573 |
|  | Prosociality |  |  |  |  | 0.09 (0.08) | 0.2682 | -0.04 (0.09) | 0.6440 |
|  | Daring |  |  |  |  | -0.15 (0.08) | 0.0569 | 0.04 (0.08) | 0.6386 |
| Cingulum | NE^1^ |  |  |  |  | 0.13 (0.06) | 0.0526 | -0.12 (0.05) | 0.0309 |
|  | Prosociality |  |  |  |  | 0.08 (0.11) | 0.4341 | 0.10 (0.10) | 0.3432 |
|  | Daring |  |  |  |  | -0.18 (0.09) | 0.0513 | -0.06 (0.10) | 0.5270 |
| Superior corona radiata | NE | -0.04 (0.10) | 0.6864 | 0.12 (0.10) | 0.2322 |  |  |  |  |
|  | Prosociality^2^ | -0.03 (0.09) | 0.7280 | **0.38 (0.12)** | **0.0022** |  |  |  |  |
|  | Daring^3^ | **-0.24 (0.07)** | **0.0008** | 0.07 (0.07) | 0.3295 |  |  |  |  |
| Superior fronto-occipital fasciculus | NE | -0.22 (0.11) | 0.0419 | 0.02 (0.10) | 0.8312 |  |  |  |  |
|  | Prosociality^4^ | -0.11 (0.11) | 0.3154 | **0.36 (0.10)** | **0.0004** |  |  |  |  |
|  | Daring | -0.12 (0.07) | 0.1147 | 0.06 (0.08) | 0.4625 |  |  |  |  |

^a^Covariates of no interest: Age in wave 1, age in wave 2, parent-classified race-ethnicity (Non-Hispanic white versus others), handedness, scanner, and TICV.

Note: β = regression coefficient; NE = negative emotionality. CADS scores and white matter tract measures converted to z-scores.

1. Significant sex-by-negative emotionality interaction after FDR correction.

2. Nominally significant sex-by-prosociality interaction (p < .0220).

3. Significant sex-by-daring interaction after FDR correction.

4. Significant sex-by-prosociality interaction after FDR correction.

Bold indicates significant after 5% false discovery rate correction for 30 tests of sex-by-disposition interactions for the specific tracts.

AAAA LOCKED SURVEYREG post hoc TBSS TICV.SAS

Table S7. Results of detailed follow-up tests in regions with significant sex-by-disposition interactions shown in Table 4 in their associations with fractional anisotropy, controlling the other dispositions and demographic covariates of no interest,^a^ separately by informant on the dispositions.

|  |  |
| --- | --- |

| CADS Informant: | | Parent | | Youth | |
| --- | --- | --- | --- | --- | --- |
| Outcome | CADS Predictor | β (SE) | P < | β (SE) | P < |
| Superior corona radiata  (Left) | NE | 0.08 (0.14) | 0.5616 |  |  |
|  | Prosociality | 0.36 (0.16) | 0.0246 |  |  |
|  | Daring | 0.27 (0.08) | **0.0017** |  |  |
| Superior corona radiata  (Right) | NE | 0.14 (0.14) | 0.3136 |  |  |
|  | Prosociality | 0.31 (0.17) | 0.0737 |  |  |
|  | Daring | 0.33 (0.11) | **0.0041** |  |  |
| Superior fronto-occipital  Fasciculus (Left) | NE | 0.22 (0.14) | 0.1303 |  |  |
|  | Prosociality | 0.43 (0.14) | **0.0031** |  |  |
|  | Daring | 0.13 (0.12) | 0.2901 |  |  |
| Superior fronto-occipital  Fasciculus (Right) | NE | 0.26 (0.16) | 0.1077 |  |  |
|  | Prosociality | 0.47 (0.17) | **0.0056** |  |  |
|  | Daring | 0.26 (0.12) | 0.0278 |  |  |
| Posterior corona radiata  (Left) | NE |  |  | -0.38 (0.12) | **0.0015** |
|  | Prosociality |  |  | -0.15 (0.13) | 0.2366 |
|  | Daring |  |  | 0.20 (0.11) | 0.0691 |
| Posterior corona radiata  (Right) | NE |  |  | -0.39 (0.12) | **0.0017** |
|  | Prosociality |  |  | -0.17 (0.12) | 0.1474 |
|  | Daring |  |  | 0.15 (0.10) | 0.1469 |
| Cingulum-Cingulate Gyrus^b^ (Left) | NE |  |  | **-0.33 (0.11)** | **0.0024** |
|  | Prosociality |  |  | 0.12 (0.16) | 0.4658 |
|  | Daring |  |  | 0.08 (0.16) | 0.6199 |
| Cingulum-Cingulate Gyrus^b^ (Right) | NE |  |  | **-0.29 (0.09)** | **0.0018** |
|  | Prosociality |  |  | 0.04 (0.15) | 0.8001 |
|  | Daring |  |  | 0.04 (0.15) | 0.7789 |
| Cingulum-Parahippocampal  (Left) | NE |  |  | -0.02 (0.12) | 0.8331 |
|  | Prosociality |  |  | -0.21 (0.13) | 0.1084 |
|  | Daring |  |  | 0.18 (0.12) | 0.1450 |
| Cingulum-Parahippocampal  (Right) | NE |  |  | -0.00 (0.10) | 0.9880 |
|  | Prosociality |  |  | -0.08 (0.15) | 0.5666 |
|  | Daring |  |  | 0.17 (0.12) | 0.1674 |

^a^Covariates of no interest: Age in wave 1, age in wave 2, parent-classified race-ethnicity (Non-Hispanic white versus others), handedness, scanner, and TICV.

^b^Subgenual and retrosplenial segments of cingulum.

Note: β = regression coefficient; NE = negative emotionality. CADS scores and white matter tract measures converted to z-scores.

Bold indicates significant after 5% false discovery rate correction in 30 tests of sex-by-disposition interactions.

CADS PREDICTION OF W2 BRAIN/AAAAA LOL190122 cingulum assym and subareas

| Table S8. Published and newly calculated associations that provide a basis for tests of mediation. | | | |
| --- | --- | --- | --- |
| Predictor | Outcome | Standardized β (S.E.) | p < |
| Prerequisite i. Dispositions Predict Psychopathology (Class et al., 2019) N = 499 | | | |
| Psychopathology Outcome: Bifactor | | | |
| *CADS Parent Ratings* | | | |
| Negative emotionality | General factor | 0.19 (0.06) | 0.002 |
| Negative emotionality | Specific externalizing | 0.15 (0.07) | 0.023 |
| Daring | Specific externalizing | 0.19 (0.07) | 0.005 |
| Prosociality | Specific externalizing | -0.18 (0.07) | 0.014 |
| *CADS Youth Ratings* | | | |
| Prosociality | General factor | -0.74 (0.25) (x sex) | 0.004 |
| Negative emotionality | Specific externalizing | -0.87 (0.24) (x sex) | 0.000 |
| Negative emotionality | Specific internalizing | 0.13 (0.06) | 0.048 |
| Daring | Specific externalizing | 0.53 (0.26) (x sex) | 0.043 |
| Psychopathology Outcome: Correlated Factors | | | |
| *CADS Parent Ratings* | | | |
| Negative emotionality | Externalizing | 0.23 (0.06) | 0.000 |
| Prosociality | Externalizing | -0.12 (0.06) | 0.040 |
| Negative emotionality | Internalizing | 0.16 (0.06) | 0.004 |
| *CADS Youth Ratings* | | | |
| Negative emotionality | Externalizing | 0.14 (0.07) | *0.051* |
| Prosociality | Externalizing | -0.21 (0.05) | *0.000* |
| Negative emotionality | Internalizing | 0.13 (0.05) | *0.008* |
| Prerequisite ii. Dispositions Predict Fractional Anisotropy (new analyses for current paper)^a^ (N = 402) | | | |
| *CADS Parent Ratings* | | | |
| Prosociality | Average FA across tracts | 0.40 (0.17) (x sex) | 0.021 |
| Daring | Average FA across tracts | 0.29 (0.11) (x sex) | 0.010 |
| Prosociality | SFOF | 0.49 (0.15) (x sex) | 0.002 |
| Daring | Superior corona radiata | 0.31 (0.10) (x sex) | 0.001 |
| *CADS Youth Ratings* | | | |
| Negative emotionality | Average FA across all tracts | -0.36 (0.10) (x sex) | 0.000 |
| Negative emotionality | Corpus callosum body | -0.53 (0.14) (x sex) | 0.000 |
| Negative emotionality | Posterior corona radiata | -0.40 (0.11) (x sex) | 0.000 |
| Negative emotionality | Cingulum | -0.26 (0.08) (x sex) | 0.003 |
| Prosociality | Corpus callosum genu | 0.25 (0.06) | 0.000 |
| Prerequisite iii. Fractional Anisotropy Predicts Psychopathology (Hinton et al., 2019)^b^  (N = 402) | | | |
| Psychopathology Outcome Model: Bifactor | | | |
| Corpus callosum body | General factor | 0.25 (0.08) | 0.001 |
| Psychopathology Outcome Model: Correlated Factors | | | |
| Average FA across tracts^b^ | Externalizing | 0.30 (0.08) (x sex) | 0.000 |
| Corpus callosum body^b^ | Externalizing | 0.20 (0.06) | 0.001 |
| Posterior corona radiata^b^ | Externalizing | 0.17 (0.06) (x sex) | 0.008 |
| Cingulum^c^ | Internalizing | 0.14 (0.07) | 0.042 |
| Cingulum^c^ | Externalizing | 0.24 (0.07) (x sex) | 0.001 |
| Corpus callosum genu^b^ | Externalizing | 0.32 (0.09) | 0.001 |

(x sex) = coefficient is significant for sex-by-disposition interaction predicting fractional anisotropy; SFOF = superior fronto-occipital fasciculus

^a^Controlling intracranial volume.

^b^New tests of association are reported Tables 3 and 4 on the present paper.


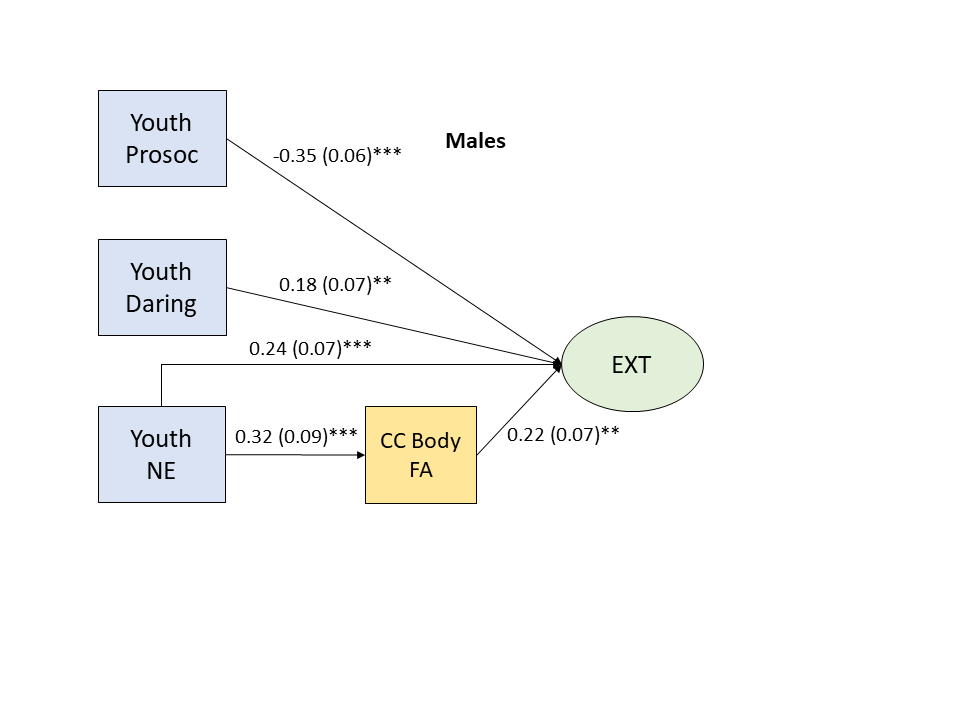

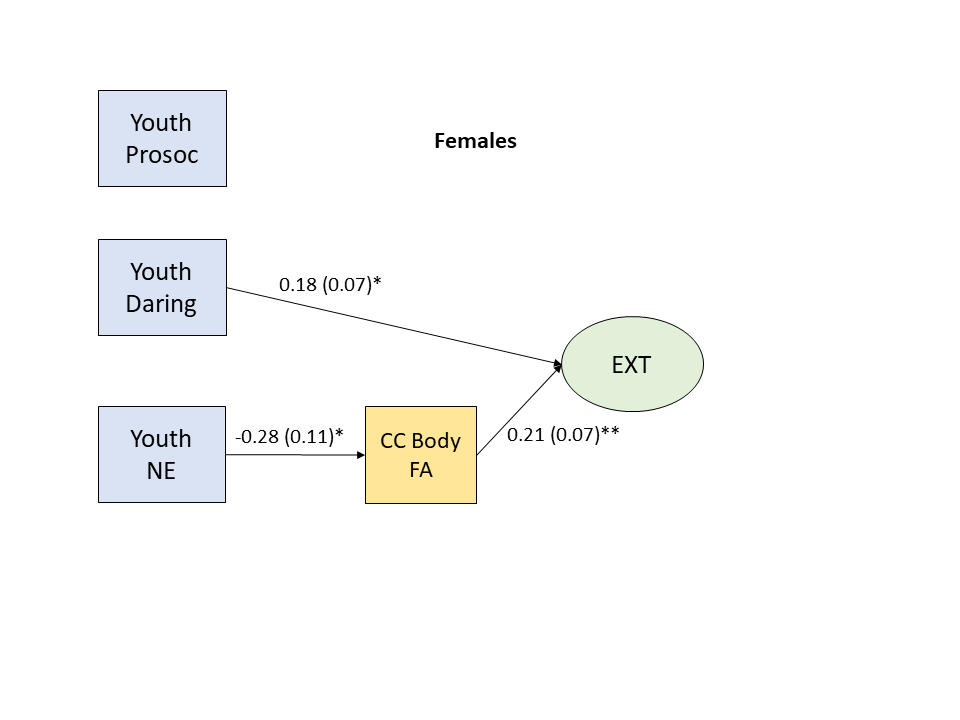


Supplemental Figure S1. Results of a multiple groups analysis to test sex-moderated direct and indirect paths between youth-rated CADS dispositions and correlated factors externalizing psychopathology mediated by FA in the body of the corpus callosum. Only paths significant at p < .05 are shown. * p < .05; ** p < .01; *** p < .001.

References for Supplement

Class, Q. A., Rathouz, P. J., Van Hulle, C. A., Applegate, B., Waldman, I. D., Zald, D. H., & Lahey, B. B. (in press). Socioemotional dispositions of children and adolescents predict general and specific second-order factors of psychopathology in early adulthood across informants: A 12-year prospective study. *Journal of Abnormal Psychology*.

Hinton, K. E., Lahey, B. B., Villalta-Gil, V., Meyer, F. A. C., Burgess, L. L., Chodes, L. K., . . . Zald, D. H. (in press). White matter microstructure correlates of general and specific second-order factors of psychopathology. . *NeuroImage: Clinical*.
